# Rituximab for relapsing nephrotic syndrome in adults: closing the evidence gap

**DOI:** 10.1093/ckj/sfag338

**Published:** 2026-09-26

**Authors:** Karol Graňák, Lisa C Willcocks

**Affiliations:** Transplant−Nephrology Department, University Hospital Martin, Martin, Slovakia; Department of I. Internal Medicine, Jessenius Faculty of Medicine of Comenius University and University Hospital Martin, Martin, Slovakia; Renal Department, Cambridge University Hospitals (NHS) Foundation Trust, Cambridge, UK

Primary nephrotic syndrome (NS) in adults encompasses a heterogeneous group of glomerular diseases, with minimal change disease (MCD) representing an important cause and typically a rapid response to high-dose corticosteroids. However, when glucocorticoids are withdrawn, a substantial proportion of patients experience frequently relapsing NS (FRNS) or steroid-dependent NS (SDNS), resulting in repeated corticosteroid exposure and considerable cumulative toxicity. This has driven the use of steroid-sparing immunosuppressive strategies, including rituximab [1]. Although rituximab has shown promising efficacy in observational studies, particularly in adults with FR/SD MCD, robust randomized evidence supporting its use in adult primary NS has been lacking [2, 3].

Isaka *et al*. now provide important evidence from a multicentre, double-blind, placebo-controlled randomized clinical trial (RCT) evaluating rituximab for relapse prevention in adults with FRNS or SDNS [4]. Sixty-six patients in remission were included in the full analysis set and received rituximab (375 mg/m² at weeks 1, 2, and 25) or placebo. The study population was predominantly composed of patients with MCD (57/66; 86%), whilst a small number of patients had focal segmental glomerulosclerosis (FSGS) (5/66; 8%) and primary membranous nephropathy (3/66; 5%). At week 49, the relapse-free rate was 87.4% with rituximab compared with 38.0% with placebo, corresponding to a hazard ratio (HR) for relapse of 0.16 (95% CI 0.05–0.46). Median time to relapse was >49 weeks versus 30.8 weeks, respectively. Only 4 out of 32 rituximab-treated patients relapsed compared with 22 out of 34 receiving placebo. Importantly, corticosteroids could be discontinued by week 49 in 71.9% of rituximab-treated patients compared with 36.4% receiving placebo (Fig. 1). Although grade 3 adverse events were more frequent with rituximab, no rituximab-related adverse events above grade 3 were observed.

**Figure 1:**
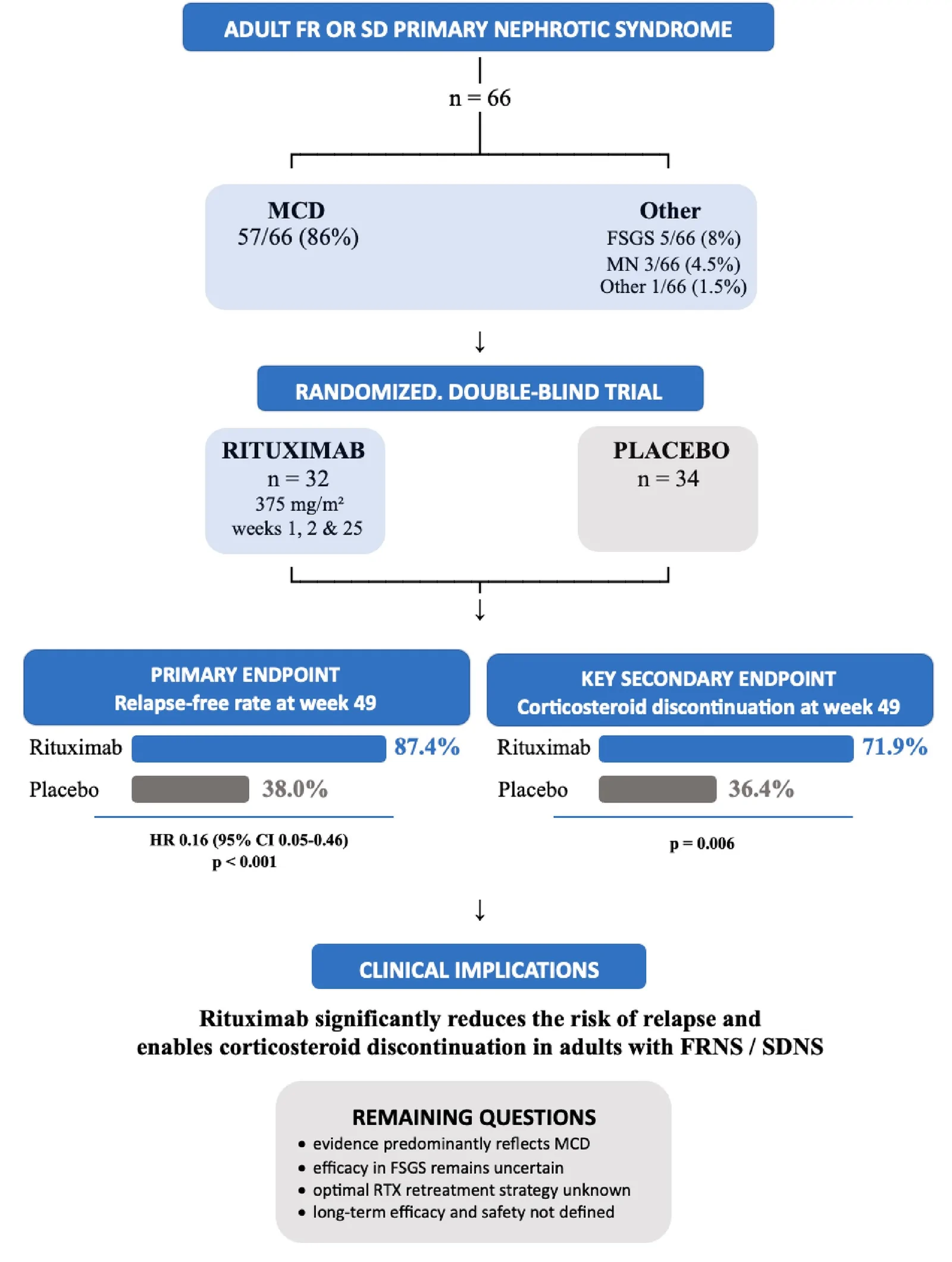
Rituximab for FRNS or SDNS in adults. Summary of the randomized, double-blind, placebo-controlled trial by Isaka *et al*. evaluating rituximab for relapse prevention in adults with primary FRNS or SDNS. Rituximab significantly improved relapse-free survival and facilitated corticosteroid discontinuation at week 49. The study population predominantly comprised patients with MCD. Abbreviations: CI, confidence interval; FRNS, frequently relapsing nephrotic syndrome; FSGS, focal segmental glomerulosclerosis; HR, hazard ratio; MCD, minimal change disease; MN, membranous nephropathy; RTX, rituximab; SDNS, steroid-dependent nephrotic syndrome.

These findings substantially strengthen the evidence supporting B-cell depletion in relapsing adult NS. Previous observational studies had already suggested that rituximab can reduce relapse frequency and immunosuppressive burden [2, 3]. More recently, long-term international data from 183 adults with difficult-to-treat MCD or FSGS have shown sustained relapse-free survival in 55% of initial responders over 3 years and suggested improved outcomes with rituximab maintenance therapy [5]. The trial by Isaka *et al*. therefore shifts rituximab from an observationally supported strategy towards one backed by randomized evidence for relapse prevention.

Several questions nevertheless remain. The trial was relatively small, conducted exclusively in Japan, and follow-up extended only si6 months beyond the final rituximab dose. The trial population predominantly had MCD: conclusions cannot be drawn for the use of rituximab in FR/SDNS secondary to FSGS and PMN. Moreover, participants were enrolled in remission; therefore, these findings establish efficacy for relapse prevention rather than remission induction. Importantly, the study does not define the optimal rituximab regimen. Whether retreatment should follow fixed intervals, B-cell repopulation or clinical relapse remains unresolved, while repeated administration raises concerns regarding cumulative infectious complications and hypogammaglobulinemia not addressed by the short trial follow-up [5, 6]. Thus, the emerging question may no longer be whether rituximab works in FRNS/SDNS secondary to MCD, but how it can best be used to achieve durable disease control while minimizing cumulative immunosuppression. Data from the TURING trial, presented at ERA Congress 2026, address some of these questions. TURING was a placebo-controlled RCT that randomized 149 patients with nephrotic FSGS (38%) and MCD (62%) as well as new-onset disease (33%) and had a median follow-up of 2.7 years. One gram rituximab given at 0, 2, and 26 weeks after starting treatment of NS significantly delayed time from remission to relapse, with a median of 23 weeks in the placebo group, whilst in the rituximab group, the median was still in remission at the end of follow up (adjusted HR 0.22, 95% CI 0.13–0.37; *P* < .0001) (Willcocks *et al*., The Lancet, in press).
